# Adverse health effects of climate change and air pollution in people with disabilities: a systematic review

**DOI:** 10.4178/epih.e2024080

**Published:** 2024-09-27

**Authors:** Nakyung Rhim, Seohyun Lee, Kyung-Hwa Choi

**Affiliations:** 1 Dankook University College of Medicine, Cheonan, Korea; 2 Department of Preventive Medicine, Dankook University College of Medicine, Cheonan, Korea; 3 Research Institute of Healthcare Bigdata, Dankook University College of Medicine, Cheonan, Korea

**Keywords:** Adverse health outcomes, Air pollution, Climate change, People with disabilities, Pollutant, Weather

## Abstract

Global warming and air pollution adversely affect the health of the entire human population, particularly older adults, people with disabilities (PWDs), and children. In this systematic review, we investigated the adverse health effects of climate change and air pollution in PWDs. We conducted a comprehensive literature search of the PubMed database using the terms “disab*,” “air pollution,” and “climate change” on July 4, 2023, and August 8, 2023 and searched the Web of Science (WOS) database on December 28, 2023. We identified 425 and 1,169 studies on climate change cited in PubMed and WOS, respectively, as well as 333 studies on air pollution in PubMed and 495 studies on air pollution in WOS. The studies were classified by type of exposure, and full-text screening was conducted to confirm that the population, intervention or exposure, comparator, outcome statement, and inclusion and exclusion criteria were met. The Newcastle-Ottawa Scale was used to assess the quality of the included cohort and case-control studies and for data analysis. In extreme temperatures, PWDs experienced higher rates of injury, heat-related illness, functional impairment, heart disease, mental disorders, and mortality than people who were non-disabled (ND). Exposure to air pollution resulted in higher rates of obesity, cardiovascular disease, poststroke neurological and functional disability, and mortality in PWDs than in people who were ND. Therefore, because PWDs were more affected by climate change and air pollution than people who were ND, sensitive policies and preparedness measures should be developed for PWDs.

## GRAPHICAL ABSTRACT

[Fig f3-epih-46-e2024080]

## Key Message

The health of the entire human population, especially vulnerable people, might be negatively impacted by climate change and air pollution. This systematic review study implies that people with disabilities (PWDs) suffer more severe physical and mental health consequences from exposure to environmental challenges compared to non-disabled individuals.

## INTRODUCTION

In recent years, the Earth has experienced a notable rise in temperature, marking an increase of approximately 1.14°C above pre-industrial era levels. This increase, as recorded over the past decade, has exceeded levels observed over the past 100,000 years [1]. Rapid climate change has created unprecedented international challenges, including severe weather events such as heatwaves, droughts, and wildfires, in addition to the spread of infectious diseases that substantially impact the global population [2].

An immediate consequence of the escalating temperatures is the rising prevalence of heat-related illnesses, which pose a considerable threat to public health. Exposure to extreme heat can exacerbate cardiovascular, respiratory, and mental disorders [3]. Older adults are currently experiencing an 85% increase in the rate of heat-related mortality compared with those between 1990 and 2000 [1].

Furthermore, climate change significantly influences the behavior and transmission patterns of infectious diseases. Factors, including temperature, humidity, and rainfall, directly affect disease vectors like the *Anopheles* mosquito, which transmits malaria. For instance, the catastrophic flooding that occurred in Pakistan in 2022 resulted in a 5-fold increase in malaria cases [4].

According to the World Health Organization (WHO), air pollution is one of the most important environmental health risks [5]. In 2019, the WHO reported that 569,000 premature mortalities were attributable to ambient air pollution. Moreover, 154,000 mortalities occurred because of household air pollution. Thirty-seven percent of outdoor air pollution-related premature mortalities resulted from ischemic heart disease and stroke, while 18%, 23%, and 11% of mortalities were due to chronic obstructive pulmonary disease, acute lower respiratory tract infections, and cancer of the respiratory tract, respectively [5].

Representative air pollutants include particulate matter (PM), sulfur oxides (SO_x_), nitrogen oxides (NO_x_), ozone (O_3_), and carbon monoxide (CO) [5]. PM is a common proxy indicator of air pollution and provides strong evidence of its negative health effects [5]. Long-term and short-term exposure to PM has been associated with morbidity and mortality resulting from cardiovascular diseases (CVD) and respiratory diseases [6].

SO_x_ are derived predominantly from the combustion of fossil fuels used in domestic heating, industry, and power generation. Exposure to SO_x_ is associated with hospitalization for asthma and emergency room visits [6]. NO_x_ are highly reactive gases commonly emitted from fuel combustion in the transportation and industrial sectors. Exposure to NO_x_ can irritate the airways and aggravate respiratory diseases. Furthermore, NO_x_ are an important precursor to O_3_, which is a pollutant closely related to the development of asthma and susceptibility to other respiratory conditions [6]. At ground level, O_3_ is one of the major components of photochemical smog. The highest O_3_ levels are typically detected during periods of sunny weather and can also be generated by household equipment, such as portable air cleaners. Exposure to excessive O_3_ can cause breathing problems, trigger asthma, reduce lung function, and result in lung disease [6]. CO is a colorless, odorless, and tasteless toxic gas produced by the incomplete combustion of carbonaceous fuels, including wood, petrol, charcoal, natural gas, and kerosene. Motor vehicles are the predominant source of CO in the ambient air. CO exposure can cause breathing difficulties, exhaustion, dizziness, various flu-like symptoms, and even life-threatening conditions [6].

Climate change and air pollution are highly interrelated factors that accelerate each other. The increased occurrence of heatwaves and dry conditions due to climate change result in elevated O_3_ concentrations. The increased incidence of wildfires amplifies the emissions of harmful greenhouse gases and PM [7]. Carbon dioxide, methane, nitrogen dioxide (NO_2_), and other emissions from human activities have augmented global warming [3]. Climate change and air pollution feed off each other, causing ecosystem disruptions that adversely affect human mental and physical health [3].

Research that involves vulnerable groups is essential from a public health perspective to assess the effects of air pollution and climate change on health. Although vulnerable individuals are subjected to the same level of exposure, they may experience greater adverse health effects because of their lower adaptive capacity [8].

In terms of environmental health, vulnerable groups include children, older adults, the socially disadvantaged, and those with chronic diseases. People with disabilities (PWDs) are people who have limitations in life activities due to a physical or mental impairment [9]. PWDs are more socially vulnerable than people who are non-disabled (ND). Moreover, PWDs have generally poorer health and higher rates of unmet medical needs [8,9]. PWDs account for 16% of the world’s population [10]. However, the criteria for recognition and the scope of support and health management for PWDs varies from country to country [11]. In Korea, as of 2019, 5.2% of the total population had registered as PWDs. Similar to Germany and Japan, Korea conducts a disability registration system to manage and support PWDs while, in cases of functional disability, most countries provide support until recovery [12].

Previous qualitative systematic reviews identified factors that were associated with climate change vulnerability and the adaptive capacity of PWDs [8]. In addition, other systematic reviews simultaneously examined climate change and air pollution, but without a particular focus on PWDs.

Therefore, in this study, we aimed to systematically review research trends regarding the adverse health effects of climate change and air pollution on PWDs by disability type and severity. The population, intervention or exposure, comparator, and outcomes (PECO) statement was set, and the elements of the PECO statement were: P, PWDs; E, climate change and/or air pollution; C, people who were ND or had a mild disability; and O, all outcomes (e.g., death, chronic disease, and emergency visits). The research question, which encompassed the PECO statement, was “What effect does exposure to climate change and air pollution have on the health of PWDs when compared to people who are ND or have a mild disability?”

## MATERIALS AND METHODS

### Search strategy

Our systematic review followed the Preferred Reporting Items for Systematic Reviews and Meta-Analyses (PRISMA) guidelines [13]. We conducted a comprehensive literature search of the PubMed database using both MeSH terms and the text words, “disab*,” “air pollution,” and “climate change” on July 4, 2023, and August 8, 2023, respectively. On December 28, 2023, we conducted a systematic search of the Web of Science (WOS) database using the same strategy as that employed for PubMed. The search terms related to air pollution and climate change used for the PubMed and WOS search strategies are listed in Table 1. We found 425 and 1,169 climate change studies cited in PubMed (from January 2000 to July 2023) and WOS (from January 2000 to December 2023), respectively, as well as 333 and 495 air pollution studies, respectively (Table 1).

Two medical school students, working independently, screened the articles by reading abstracts to select the studies. They then reviewed the full texts to obtain the penultimate list. All authors reviewed the final list of included studies. The studies were classified by exposure type, and full-text screening was conducted to confirm that the PECO statement and inclusion/exclusion criteria were met. Any discrepancies were resolved by a third reviewer, who had a doctorate in public health.

#### Inclusion criteria

We restricted our research to human studies from January 1, 2000. Only studies reported in English or Korean were considered. We only included original studies that reported a quantitative association between population health outcomes and exposure to climate change or air pollutants, using epidemiological measures of effect, such as relative risk, odds ratio, and hazard ratio. Multiple publications were available for the same study if different exposures or health outcomes were entered; therefore, duplications were excluded. We employed United States and Korean disability classifications to distinguish between PWDs and the ND. Specifically, people with diseases that could only be considered a disability in the presence of serious complications, such as high blood pressure, were not classified as PWDs; only those with diseases intrinsically considered a disability were classified as PWDs. For example, patients with asthma and allergic rhinitis are classified as PWDs by the Korean Classification of Mental and Physical Impairments [14] and the Americans with Disabilities Act (ADA) [15], and stroke is classified as a disability by the Korean Ministry of Health and Welfare and the United States Social Security Administration [16].

#### Exclusion criteria

Reviews, research letters, or commentaries were excluded. Furthermore, we excluded studies that did not include the elements of the PECO statement.

#### Data extraction

Details of each eligible study, including the first author, publication year, country in which the study had been published, study period, study design, study participants, exposure and outcome variables, relevant effect sizes and their 95% confidence intervals (CIs), and main findings were extracted and populated into Endnote version 21 (Clarivate Analytics, Philadelphia, PA, USA).

#### Quality assessment

The Newcastle-Ottawa Scale (NOS) was used to assess the quality of the included cohort and case-control studies [17]. The NOS assigns up to 9 points per study: 2 points, 4 points, and 3 points for comparability, selection, and the assessment of exposure and outcome, respectively. For data analysis in this study, we calculated the sum of each item score, with scores ≥ 7 representing high quality and scores < 7 representing low quality.

### Ethics statement

The study consisted of a systematic review employing secondary data. The study protocol was approved by the Institutional Review Board of Dankook University (IRB No. DKU 2023-04-004-004). The need for informed consent was waived by the same board.

## RESULTS

### Literature search results

**Figure 1.**
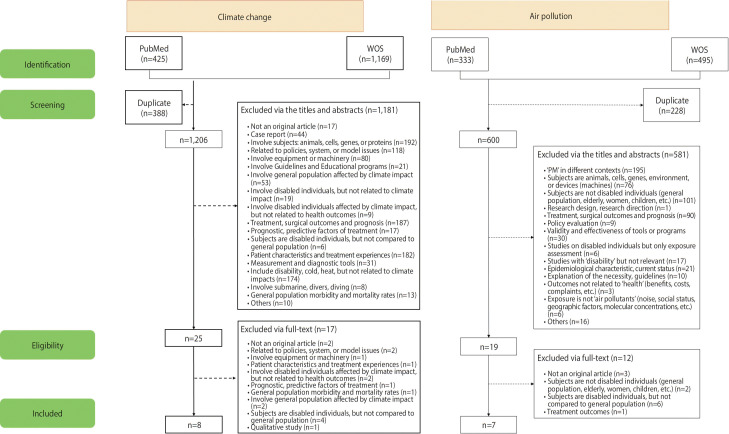
Selection process for the studies included in the systematic review. Health outcomes in people with disabilities due to climate change and air pollution were screened and selected. PM, particulate matter; WOS, Web of Science.

The selection process for the studies included in our systematic review is presented in Figure 1. Of the 1,594 climate change studies, 388 were duplicate studies, 1,231 did not meet the PECO criteria or matched the exclusion criteria (i.e., non-original articles, case report articles, articles addressing health effects in the general population, articles addressing treatment outcomes using heat, and articles on completely different topics). Consequently, these studies were excluded. Of the 828 air pollution studies, 228 were duplicate studies, and 619 did not meet the PECO criteria or did match the exclusion criteria and were subsequently excluded. Articles were also excluded if the subject was not PWDs, the exposure variable was not an air pollutant, the search term “PM” was used to define a term other than “particulate matter,” and the article presented a status survey or guideline, among others.

Finally, 8 climate change studies were selected, including 1 case-control [18], 2 cohort [19,20], 3 cross-sectional [21-23], 1 ecological [24], and 1 case-crossover [25]. Seven air pollution studies were selected, including 4 cohort studies (including national longitudinal studies) [26-29], 1 cross-sectional study [30], and 2 case-crossover studies [31,32]. The quality of the selected articles, as assessed using the NOS, is presented in Table 2. The scores of all cohort studies were ≥ 7 points. The non-response rate was investigated in case-control or case-crossover studies.

### Summary of the studies

A summary of the selected studies addressing the adverse health effects of climate change and air pollution on PWDs is shown in Table 3. PWDs included individuals with and without an underlying disease. PWDs without disease conditions included children with disabilities [18], older adults requiring assistance in daily activities [20], individuals with various disabilities [22-25,32], older adults with disabilities [28,30], and Medicaid beneficiaries [31]. PWDs also included nursing home residents with severe disabilities [19], individuals with asthma and allergic rhinitis [21], people with chronic disabling diseases [26], and patients with stroke [27,29].

In the 8 studies that addressed the adverse health effects of climate change on PWDs, the exposure variables were extreme temperatures or extreme weather events. These 8 studies were organized by the type of exposure: “heatwave” [19,20,24], “non-optimal temperatures, such as extreme heat or cold” [25], “winter (October to March)” [18], “cold weather in Finland” [21], and “winter storm Uri” [22,23] (Table 3).

In the 7 studies that addressed the adverse health effects of air pollution on PWDs, the exposure variables were fine particulate matter (PM_2.5_, PM_10_), SO_2_, NO_2_, O_3_, CO, NO_x_, sulfate, nitrate, ammonium, organic matter (OM), and black carbon (BC). One study investigated protective factors, such as residential greenness. These 7 studies were categorized according to the exposure variables, with 2 and 3 studies investigating PM [31,32] or PM and other pollutants [26,28,30], respectively, and 1 study each addressing O_3_ [27], OM [29], and BC [29]. Some of these studies used the Air Quality Index [30] or residential greenness [28] as exposure variables (Table 3).

### Main results of the systematic review

The adverse health effects of climate change on PWDs due to hot weather included mortality [19,20,24], heat-related health outcomes (HRHOs) [20], and hospitalization for CVD [25], whereas those due to cold weather included injury [18], functional disability [21], exacerbation of health problems [21], post-traumatic stress (PTS) [22], anxiety and depression [23], and hospitalization for CVD [25] (Table 4). During the 2003 heatwave in Paris, France, the mortality rate among PWDs in nursing homes was 2.14 times (95% confidence interval [CI], 1.10 to 4.17) higher in those with respiratory insufficiency than in the control group with other disabilities [19]. HRHOs, such as emergency department visits, hospitalizations, and mortalities on hot days with maximal temperatures ≥ 30°C occurred 2.19 times (95% CI, 1.03 to 4.67) more frequently among older adults requiring assistance with activities of daily living than among the general population [20]. Men and women patients with both asthma and allergic rhinitis were 4.02 (95% CI, 2.89 to 5.59) and 4.60 (95% CI, 3.69 to 5.73) times more likely, respectively, to experience exacerbations of health problems during cold weather than the controls [21]. In winter storms, PWDs were 4.40 (95% CI, 2.71 to 7.14), 6.91 (95% CI, 3.26 to 14.67), and 6.01 (95% CI, 2.62 to 13.80) times more likely to suffer from PTS, anxiety, and depression, respectively, than were people who were ND [22,23].

Regarding studies on the adverse health effects of air pollution on PWDs, the health outcomes included the 30-day hospital mortality rate [26], obesity and abdominal obesity [30], CVD [31,32], poststroke neurological and functional disability [27,29], and mortality [28] (Table 5). One study reported a significantly higher risk of hospital admission for CVD due to PM in individuals with brain lesions [32]. Most studies reported that the risk of health effects was higher in people with severe disability than in those with mild disabilities or the ND. For every 10 µg/m3 increase in particulate matter less than 10 μm in diameter (PM10), the hospitalization rate for CVD in patients with mild disabilities increased by 1.40% (95% CI, -0.20 to 2.90), while the hospitalization rate for CVD in patients with severe disabilities increased by 3.00% (95% CI, 0.90 to 5.00) [32].

### Mapping of the systematic review

**Figure 2.**
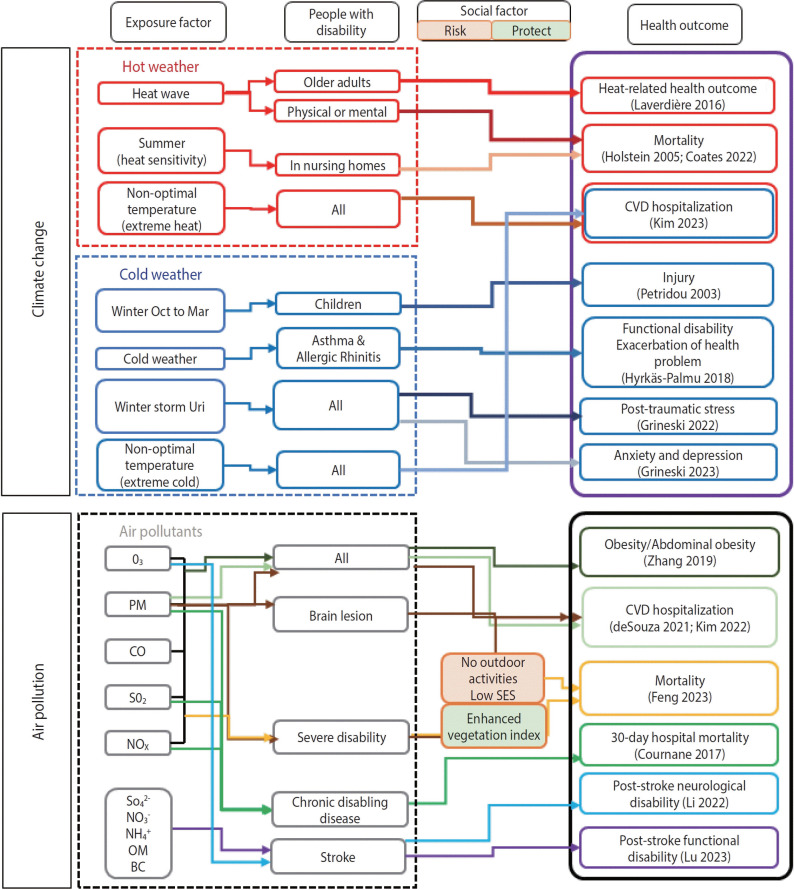
Mapping of the systematic review. Health outcomes in people with disabilities due to climate change and air pollution are depicted. Each paper is represented by a different colored arrow. O_3_, ozone; PM, particulate matter; CO, carbon monoxide; SO_2_, sulfur dioxide; NO_x_, nitrogen oxides; SO_4_^2-^, sulfate; NO_3_^-^, nitrate; NH_4_^+^, ammonium; NO_2_, nitrogen dioxide; OM, organic matter; BC, black carbon; SES, social economic status; CVD, cardiovascular disease.

The adverse health outcomes of PWDs due to climate change and air pollution are depicted in a map in Figure 2. The exposure factors for climate change were divided into hot and cold weather and into air pollutants, such as PM, CO, and SO_2_. PWDs were categorized into cohorts according to the type and severity of their disability. Social factors included risks that worsened health and protective factors that prevented health deterioration. Rather than categorize each study separately, the studies that addressed the same adverse health outcomes were grouped together.

## DISCUSSION

PWDs experienced greater adverse health effects than people who were ND when exposed to climate change and air pollution. Furthermore, differences in adverse health effects were observed based on the severity or type of disability.

To the best of our knowledge, no prior systematic review has identified the adverse health effects of climate change and air pollution on PWDs. Although one systematic review summarized the association between specific diseases and air pollution, it was not restricted to PWDs. It included reports regarding disease exacerbations and mortalities in PWDs and studies on disease onset in people who were ND [33]. In addition, the study did not address climate change. Gaskin et al. [8] conducted a systematic qualitative review of the adverse health effects of climate change. Although a few systematic reviews have addressed climate change and air pollution simultaneously, with some focusing on pregnant women or children, none have focused on PWDs. Unlike previous studies, the present study distinguished between PWDs and people who were ND when examining the adverse health effects of environmental changes on the general population. Our study is the first to focus exclusively on PWDs and examine their disease progression and mortality in response to environmental exposure.

We could not perform a meta-analysis due to the variability in exposure among studies. However, we were able to map the adverse health outcomes in PWDs due to climate change and air pollution. Because our study addressed both climate change and air pollution, as opposed to a single public health concern, it is a practical contribution towards developing policies and interventions in the face of recent environmental challenges. Importantly, we only selected studies on the adverse health effects in PWDs, a particularly vulnerable group, rather than the general population. Summarizing these studies confirmed that the health outcomes of PWDs were more vulnerable to climate change and air pollution than those of people who were ND. Furthermore, we demonstrated adverse health outcomes according to the type and severity of disabilities. People with physical or mental disabilities had higher mortality and hospitalization rates in hot weather than people without disabilities, and the frequency of mental illness or existing conditions was more severe in cold weather. People with disabilities due to brain lesions had higher mortality, hospitalization, and functional impairment rates due to air pollutants than people without disabilities, with more severe disabilities leading to more severe health outcomes.

Our study found that PWDs were more affected by environmental changes, such as climate change and air pollution, than people who were ND. People with brain lesions were at higher risk of heart disease due to exposure to fine PM than those with other types of disability [32]. Moreover, people with severe disabilities were found to be more affected than those with milder disabilities throughout all studies. Thus, differences existed in the prevalence and mortality rates of an illness caused by exposure to air pollution based on the type or severity of the disability. However, these effects can vary according to social factors, such as socioeconomic status and access to outdoor activities; therefore, the results were not consistent in some disability types. During a 2003 heat wave in France, people with epilepsy, terminal renal insufficiency, or a psychotic state showed a high risk of mortality, but the increase was not statistically significant [19], and those with cognitive impairment had a high risk for HRHOs [20]. Moreover, all PWDs had a lower (non-significant) risk of hospitalization for CVD [25]. Only people with asthma, but without allergic rhinitis, had a higher (non-significant) rate of cold-related functional disability [21]. Despite the limited number of studies, the results regarding extreme weather imply that PWDs are more affected by cold weather than hot weather. A study regarding air pollutants (i.e., PM10) found a non-significant risk of adverse health effects in those with mild physical, vision, and hearing disabilities [32]. These findings could be attributed to (1) some disabilities were medically sensitive to external environmental factors while others were not, (2) differences in the way people with certain disabilities cope with climate change and air pollution, or (3) the level of social care received. Extreme weather may disrupt access to healthcare services, medications, oxygen, hemodialysis, personal care assistance, and medical devices [34]. PWDs who are more vulnerable to deficits in these services may be more affected by environmental changes than people with other disabilities.

This study had several limitations. First, although extensive, our search included articles in PubMed and WOS only. Second, only reports in English or Korean were selected. Third, no meta-analysis was performed. Therefore, further research is required to investigate the effect of climate change on health in particularly vulnerable populations. Based on this research, sensitive policies and preparedness measures for PWDs should be developed.

## CONCLUSION

Through this systematic review study, it has been demonstrated that PWDs experience more severe physical and mental health impacts from exposure to climate change and air pollution compared to ND. This implies the need for targeted interventions and policies to protect vulnerable populations, such as PWDs, in the face of environmental challenges.

## Figures and Tables

**Figure f3-epih-46-e2024080:**
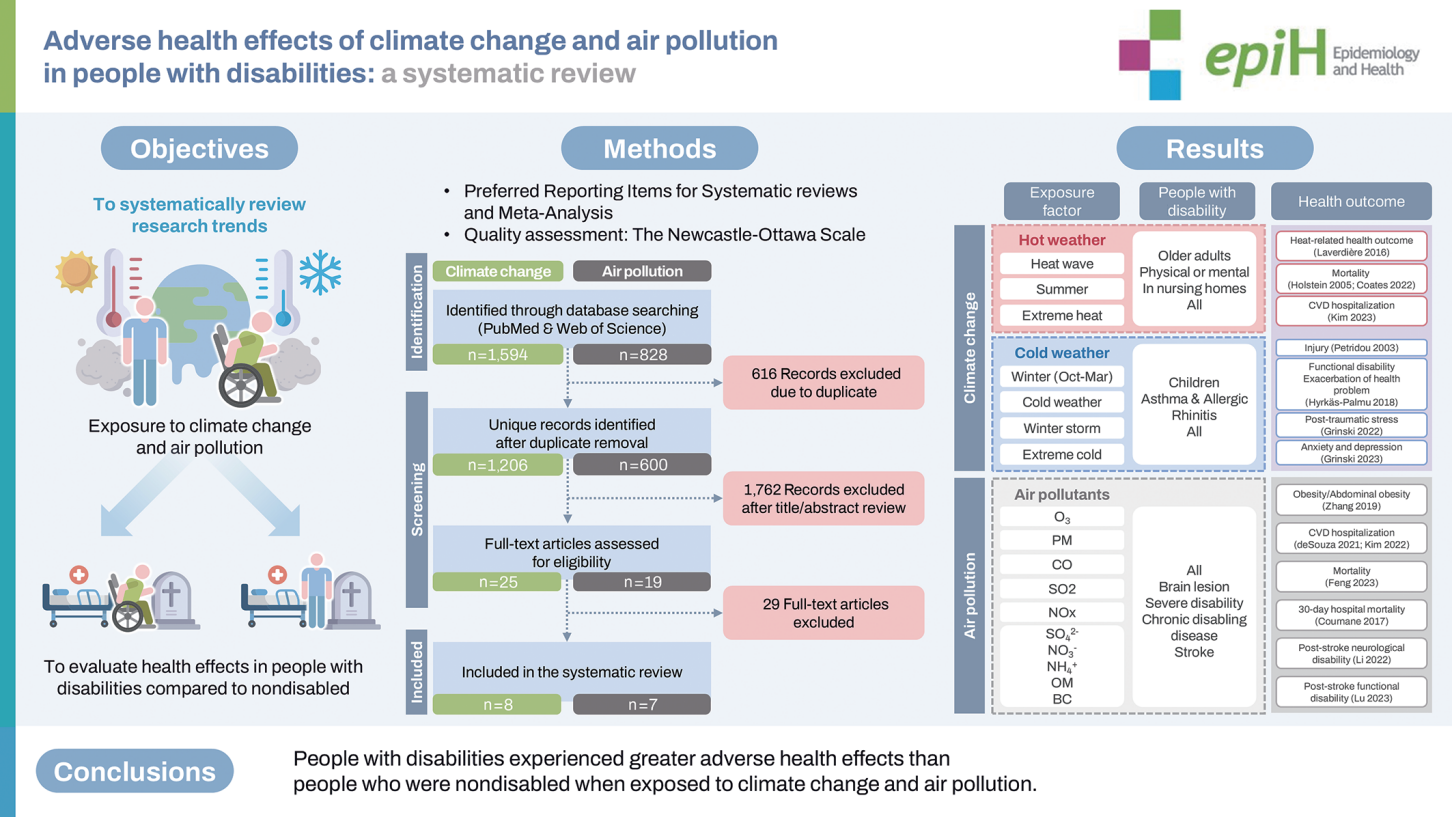


**Table 1.** Search terms by type of exposure and source database used in a systematic review of health outcomes in people with disabilities exposed to climate change and air pollution

Exposure	Source database	
	PubMed	Web of Science
Climate change	(‘disabled’[Title/Abstract] or ‘disability’[Title/Abstract]) & (‘heat’[Title/Abstract] OR ‘cold’[Title/Abstract] OR ‘climate change’[Title/Abstract]) AND (humans[Filter]) AND (english[Filter] OR korean[Filter]) AND (2000:2023[pdat]) NOT (review[Publication Type]) NOT (systematic review[Publication Type]) NOT (DALY*[Title/Abstract]) NOT (randomized clinical trial [Publication Type])	(AB=(disab*)) AND (AB=(heat) OR AB=(cold) OR AB=(climate change)) AND ((LA=English) or LA=(Korean)) and (DT=(article)) not (AB=(burden)) not (AB=DALY*) not (AB=(disability-adjusted life years)) not (TI=review) not (TI=device) not (TI=trial)
Air pollution	(disab*[Title/Abstract] AND (“particulate matter”[Title/Abstract] OR “pm”[Title/Abstract] OR “ozone”[Title/Abstract] OR “air pollutant”[Title/Abstract] OR “air pollution”[Title/Abstract] OR “sulfur oxide”[Title/Abstract] OR “nitrogen oxide”[Title/Abstract]) AND (english[Filter] OR korean[Filter]) AND (2000:2023[pdat]) AND (humans[Filter]) NOT (review[Publication Type]) NOT (systematic review[Publication Type]) NOT (DALY*[Title/Abstract]) NOT (clinical trial[Publication Type]))	(AB=disab*) AND (AB=(particulate matter) OR AB=(pm) OR AB=(ozone) OR AB=(air pollutant) OR AB=(air pollution) OR AB=(sulfur oxide) OR AB=(nitrogen oxide)) AND ((LA=english) OR LA=korean) and (DT=(article)) not (AB=(burden)) not (AB=DALY*) not (AB=(disability-adjusted life years)) not (TI=trial)

**Table 2.** Distribution of the Newcastle-Ottawa Scale quality assessment of articles in a systematic review of environmental effects on the health of people with disabilities

Categories	Items	Climate change (score)		Air pollution (score)	
		All	Awarded	All	Awarded
All					
Score (Min-Max)		9	6-7	9	8-9
Case-control study/case cross-over design					
Selection	Is the case definition adequate?	6	6	3	3
	Representativeness of the cases	6	5	3	3
	Selection of controls	6	5	3	3
	Definition of controls	6	6	3	3
Comparability	Comparability of cases and controls on the basis of the design or analysis	12	7	6	3
Exposure	Ascertainment of exposure with record	6	6	3	2
	Same method of ascertainment for cases and controls	6	6	3	3
	Non-response rate	6	0	3	0
Cohort study					
Selection	Representativeness of the exposed cohort	2	2	4	3
	Selection of the non-exposed cohort	2	2	4	4
	Ascertainment of exposure	2	2	4	4
	Demonstration that outcome of interest was not present at start of study	2	2	4	4
Comparability	Comparability of cohorts on the basis of the design or analysis	4	4	8	8
Outcome	Assessment of outcome	2	2	4	4
	Was follow-up long enough for outcomes to occur	2	2	4	3
	Adequacy of follow-up of cohorts	2	1	4	4

Min, minimum; Max, maximum.

**Table 3.** Summary of the studies in the systematic review of environmental effects on the health of persons with disabilities

Study	Country	Study design/duration	Study subject	Exposure	Comparator	Health outcome
Climate change						
Petridou et al., 2003 [18]	Greece	Case-control study/1996-2000	Children with disability	Winter (Oct to Mar)	Non-disabled children	Injury
Holstein et al., 2005 [19]	France	Cohort study/2000-2003	Nursing home residents with severe disabilities	Heat wave	Nursing home patients with mild disabilities	Mortality
Laverdière et al., 2016 [20]	Canada	Cohort study/2006-2010	Older adults requiring assistance in activities of daily living	Heat wave	Older adults	Heat-related health outcome
Hyrkäs-Palmu et al., 2018 [21]	Finland	Cross-sectional study/2007 and 2012	Patients with asthma and allergic rhinitis	Cold weather in Finland	No asthma or allergic rhinitis	Functional disability, exacerbation of health problem
Coates et al., 2022 [24]	Australia	Ecological study/2001-2018	Presence of disabilities (physical or mental)	Heat wave (2009 and 2014)	Non-disabled	Mortality
Grineski et al., 2022 [22]	USA	Cross-sectional study/2021	People with disabilities	Winter storm Uri	Non-disabled	Post-traumatic stress
Grineski et al., 2023 [23]	USA	Cross-sectional study/2021	People with disabilities	Winter storm Uri	Non-disabled, not impacted by Uri	Anxiety and depression
Kim et al., 2023 [25]	Korea	Case-crossover design/2002-2019	People with disabilities	Non-optimal temperatures (extreme heat, extreme cold)	Non-disabled	Hospitalization for cardiovascular disease
Air pollution						
Cournane et al., 2017 [26]	Ireland	Cohort study/2002-2015	People with chronic disabling disease (score=4)	PM_10_, SO_2_, NOx	People with chronic disabling disease (score=1, 2, 3)	30-day hospital mortality
Zhang et al., 2019 [30]	China	Cross-sectional study/2015	Individuals aged 60 yr or older with disabilities	PM_2.5_, PM_10_, SO_2_, NO_2_, O_3_, CO	Individuals aged 60 yr or older without disabilities	Obesity and abdominal obesity
deSouza et al., 2021 [31]	USA	Time-stratified case-crossover design/2000-2012	Medicaid beneficiary	PM_2.5_	Medicare enrollee not eligible for Medicaid	Cardiovascular disease
Kim et al., 2022 [32]	Korea	Time-stratified case-crossover design/2002-2015	People with disabilities	PM_10_, SO_2_, NO_2_, O_3_, CO	Severity and type of disability	Cardiovascular disease
Li et al., 2022 [27]	China	National longitudinal study/2013-2019	Patients with stroke	O_3_	Stroke (change in modified ranking scale)	Poststroke neurological disability
Feng et al., 2023 [28]	China	Cohort study/2017-2021	Individuals aged 60 yr or older with severe disabilities	Residential greenness (mediator: PM_2.5_, NO_2_, CO, SO_2_, O_3_)	Individuals aged 60 yr or older with mild disabilities	Mortality
Lu et al., 2023 [29]	China	National multicenter longitudinal study/2013-2019	Patients with stroke	Five components (SO_4_^2-^, NO_3_^-^, NH_4_^+^, organic matter, and black carbon)	Stroke (change in modified ranking scale)	Poststroke functional disability

PM, particulate matter; O_3_, ozone; NO, nitrous oxide; NH_4_^+^, ammonium; SO_4_^2-^, sulfate; NO_3_^-^, nitrate; CO, carbon monoxide; SO_2_, sulfur dioxide; NO_2_, nitrogen dioxide; PM_2.5_, particulate matter smaller than 2.5 μm in diameter; PM_10_, particulate matter less than 10 μm in diameter.

**Table 4.** Health outcomes due to climate change among people with disabilities: results of a systematic review

Study	Risk factor	Health outcome	Study subject		Type of effect	Effect size	95% CI		Exposure level/reference
							LL	UL	
Hot weather									
Holstein et al., 2005 [19]	Heat wave in 2003	Mortality	Disabled patients in nursing homes	Respiratory insufficiency vs. others	Mortality rate ratio	4.74	3.03	7.42	Before heat wave
						2.14	1.10	4.17	Heat wave
						1.20	0.53	2.69	After heat wave
				Dementia vs. others		1.17	0.85	1.63	Before heat wave
						0.89	0.66	1.21	Heat wave
						1.51	1.10	2.07	After heat wave
				Parkinson disease vs. others		1.43	0.84	2.42	Before heat wave
						1.10	0.61	1.98	Heat wave
						2.04	1.33	3.14	After heat wave
				Terminal renal insufficiency vs. others		1.46	0.65	3.28	Before heat wave
						1.28	0.53	3.12	Heat wave
						1.66	0.82	3.36	After heat wave
				Epilepsy vs. others		1.28	0.60	2.72	Before heat wave
						1.27	0.60	2.70	Heat wave
						1.43	0.74	2.79	After heat wave
				Psychotic states vs. others		0.93	0.64	1.33	Before heat wave
						0.93	0.65	1.34	Heat wave
						1.13	0.83	1.54	After heat wave
				Severe or complex chronic disease vs. others		2.52	1.86	3.41	Before heat wave
						1.34	1.03	1.75	Heat wave
						1.60	1.26	2.04	After heat wave
Laverdière et al., 2016 [20]	Hot day (maximal temperature ≥30°C) between 2006 and 2010	Heat-related health outcome: emergency department presentations, hospitalization, or mortality	Older adults living in Quebec	Needs help in activities of daily living	Odds ratio	2.19	1.03	4.67	
				Cognitive impairment		1.22	0.23	6.53	
Coates et al., 2022 [24]	Heat wave	Mortality with heart problem	Presence of physical disabilities		Fatality ratio	2.9	-	-	Vs. non-disabled
		Mortality with respiratory problems				0.8	-	-	
		Mortality with obesity				0.4	-	-	
		Mortality with kidney disease				0.4	-	-	
		Mortality with mobility problems				0.3	-	-	
		Mortality with diabetes				0.3	-	-	
		Mortality with cerebrovascular disease				0.1	-	-	
		Mortality with others				1.5	-	-	
Kim et al., 2023 [25]	Non-optimal temperatures (extremely hot, 99th percentile)	Hospitalization for cardiovascular disease	People with disability		Relative risk	0.99	0.87	1.12	20ºC as reference
			Non-disabled			1.07	0.99	1.15	
Cold weather									
Petridou et al., 2003 [18]	Cold weather (Oct-Mar)	Injury	Disabled children vs. non-disabled children		Odds ratio	1.37	1.06	1.75	Apr-Sep as reference
Hyrkäs-Palmu et al., 2018 [21]	Cold weather	Cold-related functional disability	Asthma with allergic rhinitis	Men	Prevalence ratio	1.16	0.90	1.50	Vs. no asthma or allergic rhinitis
	Helsinki -6.8 to 17.7ºC			Women		1.40	1.12	1.76	
			Asthma without allergic rhinitis	Men		1.29	0.93	1.80	
	Sodankylä -15.8 to 13.9°C			Women		1.36	0.92	2.02	
		Exacerbation of health problem	Asthma with allergic rhinitis	Men		4.02	2.89	5.59	
				Women		4.60	3.69	5.73	
			Asthma without allergic rhinitis	Men		4.28	2.88	6.36	
				Women		3.77	2.67	5.34	
Grineski et al., 2022 [22]	Winter storm Uri	Post-traumatic stress	People with disability		Odds ratio	4.40	2.71	7.14	Vs. non-disabled
Grineski et al., 2023 [23]	Winter storm Uri	Anxiety	People with disability and impacted by Uri		Odds ratio	6.91	3.26	14.67	Vs. non-disabled and not impacted by Uri
		Depression				6.01	2.62	13.80	
Kim et al., 2023 [25]	Non-optimal temperatures (extreme cold)	Hospitalization for cardiovascular disease	People with disability		Relative risk	1.22	1.13	1.32	30th percentile vs. 20ºC
						1.11	1.01	1.21	5th percentile vs. 20ºC
			Non-disabled			1.09	0.97	1.23	1st percentile vs. 20ºC

LL, lower limit; UL, upper limit.

**Table 5.** Health outcomes due to air pollution exposure among people with disabilities: results of a systematic review

Study	Exposure	Outcome	Subjects	Type of effect	Effect size	95% CI		Unit increase/exposure level
Cournane et al., 2017 [26]	PM_10_	30-day hospital mortality	People with chronic disabling disease - low	Mortality (%)	10.8	-	-	Quintile 1
					16.9	-	-	Quintile 5
			People with chronic disabling disease - high		11.6	-	-	Quintile 1
					22.2	-	-	Quintile 5
Zhang et al., 2019 [30]	AQI	Obesity	People with disabilities	Odds ratio	1.27	1.02	1.57	Interaction term (AQI*disability)
		Abdominal obesity			1.27	1.06	1.54	
deSouza et al., 2021 [31]	PM_2.5_	Hospitalization rates for CVD	Adults receiving Medicaid (low income and disabled)	% change	0.90	0.60	1.10	10 µg/m^3^
			Adults not receiving Medicaid		0.80	0.60	0.90	
Kim et al., 2022 [32]	PM_10_	Hospitalization rates for CVD	Disability	% change				10 µg/m^3^ at lag03
			Non-disabled		0.00	-0.50	0.50	
			People with disabilities		1.90	0.70	3.20	
			Type of disability					
			Physical		1.40	-0.60	3.40	
			Brain lesion		2.70	0.50	5.00	
			Visual		3.00	-1.00	7.10	
			Hearing		1.60	-0.25	5.90	
			Others		1.30	-1.90	4.60	
			Severity					
			Mild		1.40	-0.20	2.90	
			Severe		3.00	0.90	5.00	
Li et al., 2022 [27]	O_3_	Increased mRS score vs. unchanged or decreased mRS score	Patients with stroke	Odds ratio	1.23	1.09	1.37	10 µg/m^3^ (peak season)
					1.28	1.09	1.52	10 µg/m^3^ (annual mean)
Feng et al., 2023 [28]	Enhanced vegetation index within the 500 m buffer zone (EVI500m)	Mortality	Mild–moderate disability	Hazard ratio	Reference			(0.00, 0.27)
					0.86	0.80	0.91	(0.27, 0.31)
	Mediator: PM_2.5_, NO_2_, CO, SO_2_, and O_3_				0.73	0.65	0.81	(0.31, 0.38)
					0.63	0.55	0.75	(0.38, 0.72)
			Severe disability		Reference			(0.00, 0.27)
					0.95	0.92	0.97	(0.27, 0.31)
					0.89	0.85	0.94	(0.31, 0.38)
					0.86	0.80	0.91	(0.38, 0.72)
Lu et al., 2023 [29]	Organic matter	Poststroke functional disability (change in mRS scores)	Patients with stroke	Point increase	0.062	0.013	0.111	Interquartile range
	Black carbon				0.012	-0.030	0.053	
	NO_3_^-^				-0.002	-0.075	0.071	
	NH_4_^+^				0.008	-0.056	0.072	
	SO_4_^2-^				0.057	0.003	0.112	

AQI, Air Quality Index; CVD, cardiovascular disease; mRs, modified ranking scale; PM, particulate matter; O_3_, ozone; NO, nitrous oxide; NH_4_^+^, ammonium; SO_4_^2-^, sulfate; NO_3_^-^, nitrate; CO, carbon monoxide; SO_2_, sulfur dioxide; NO_2_, nitrogen dioxide; PM_2.5_, PM smaller than 2.5 μm in diameter; PM_10_, PM less than 10 μm in diameter.
